# Eliminating Halogen Vacancies Enables Efficient MACL‐Assisted Formamidine Perovskite Solar Cells

**DOI:** 10.1002/advs.202306280

**Published:** 2023-12-08

**Authors:** Zhiyong Liu, Tianxiao Liu, Meng Li, Tingwei He, Gaofu Guo, Pengfei Liu, Ting Chen, Jien Yang, Chaochao Qin, Xianqi Dai, Mingjian Yuan

**Affiliations:** ^1^ School of Physic Henan Key Laboratory of Photovoltaic Materials Henan Normal University Xinxiang 453007 China; ^2^ Key Lab for Special Functional Materials of Ministry of Education National & Local Joint Engineering Research Center for High‐efficiency Display and Lighting Technology School of Materials Science and Engineering and Collaborative Innovation Center of Nano Functional Materials and Applications Henan University Kaifeng 475004 China; ^3^ College of Physics Science and Technology Hebei University Baoding 071002 China; ^4^ Department of Chemistry Nankai University Tianjin 300071 China

**Keywords:** perovskite solar cell, methylammonium chloride, crystallization kinetic, high‐performance, defect passivation

## Abstract

Methylammonium chloride (MACl) additive is almost irreplaceable in high‐performance formamidine perovskite photovoltaics. Nevertheless, Some of the problems that can arise from adding MACl are rarely mentioned. Herein, it is proposed for the first time that the addition of MACl would cause the non‐stoichiometric ratio in the perovskite film, resulting in the halogen vacancy. It is demonstrated that the non‐synchronous volatilization of methylamine cations and chloride ions leads to the formation of halogen vacancy defects. To solve this problem, the NH_4_HCOO is introduced into the perovskite precursor solution to passivate the halogen vacancy. The HCOO^−^ ions have a strong force with lead ions and can fill the halogen vacancy defects. Consequently, the champion devices' power conversion efficiency (PCE) can be improved from 21.23% to 23.72% with negligible hysteresis. And the unencapsulated device can still retain >90% of the initial PCE even operating in N_2_ atmosphere for over 1200 h. This work illustrates another halogen defect source in the MACl‐assisted formamidine perovskite photovoltaics and provides a new route to obtain high‐performance perovskite solar cells.

## Graphical abstract

The mechanism of MACl‐assisted crystallization on high‐performance formamidine perovskite photovoltaics is not fully understood, especially the introduction of methylamine cations. The addition of MACl would cause the non‐stoichiometric ratio in perovskite film, then the non‐synchronous volatilization of methylamine cations and chloride ions would lead to the formation of halogen vacancy defects. The pseudo‐halogen passivation method shows better performance, which emphasizes the improvement effect of this method on perovskite devices.

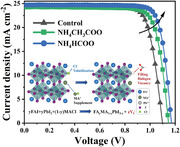

## 1 Introduction

Perovskite solar cells (PSCs) have achieved a rapid growth of power conversion efficiency (PCE) from 3.8% to 26.1%.^[^ ^1^, ^2^, ^3^, ^4^ ^]^ High‐performance solar cells usually employ formamidinium lead triiodide (FAPbI_3_) material as the light absorption layer. It is a common strategy that methylammonium chloride (MACl) additives assist crystallization to form stable *α*‐phase FAPbI_3_.^[^ ^5^, ^6^, ^7^ ^]^ It has already been confirmed that over 5 mol.% MA^+^ can go into the perovskite lattice after introducing >30 mol.% MACl additives. While the introduction of MA components reduces the stability of the resulting perovskite films, especially under thermal conditions.^[^ ^8^, ^9^ ^]^ Some strategies have been used to prepare MA‐free perovskite films, such as dimensional engineering,^[^ ^10^ ^]^ crystallization regulation,^[^ ^11^ ^]^ etc. Unfortunately, the resulting devices exhibit inferior PCE compared to MACl‐assisted formamidinium perovskite devices. It follows that MACl additive plays an irreplaceable role in high‐performance PSCs. Nevertheless, The PCE of MACl‐assisted device has not reached its theoretical limit. It suggests that there are still some defects (V_I_^−^, V_Pb_^2+^, Pb_I_ etc.) in perovskite that inhibit the further enhancement of PCE.

Among these defects, halogen ions are easy to form vacancies due to their low defect‐forming energy and high carrier mobility.^[^ ^12^, ^13^, ^14^, ^15^, ^16^ ^]^ These vacancy defects will act as non‐radiative recombination centers to degrade the performance of PSCs. Because pseudo‐halogens have similar ionic radii to halogens, they can directly fill the resulting halogen vacancy and thus make the perovskite structure more stable. At the same time, some pseudo‐halogen ions with unique chemical properties, have the passivation effect and can regulate the crystallization process of perovskite. Therefore, a lot of work has been done to passivate halide vacancies using pseudo‐halogen ions (such as SCN^−^, OCN^−^, and HCOO^−^)^[^ ^17^, ^18^, ^19^ ^]^ to prepare high‐performance PSCs. Inspired by these works, we designed some pseudo‐halogen anions to solve the halogen vacancy issues caused by MACl evaporation.

In this work, we first put forward that the addition of MACl leads to a non‐stoichiometric ratio of perovskite, and the resulting perovskite would inevitably produce halogen vacancies. Two pseudo‐halogen anions, acetate anion (CH_3_COO^−^), and formate anion (HCOO^−^), were chosen to passivate the halogen defects of perovskite films. The DFT calculations and experiments together demonstrate that both HCOO^−^ and CH_3_COO^−^ can fill V_I_^−^ and bind to Pb^2+^, further passivate V_I_^−^ and stabilize Pb^2+^ on the perovskite surface. Moreover, the doping of HCOO^−^ and CH_3_COO^−^ significantly improved the crystallization quality of perovskite film, and the halogen defects have obviously been inhibited. The femtosecond transient absorption spectra are also employed to show meticulously that HCOO^−^ modified perovskite films greatly reduce the non‐radiative recombination caused by defect sites. Eventually, the PCE of the champion device was increased from 21.32% to 23.72%, showing negligible hysteresis. And the unencapsulated device can still retain >90% of the initial PCE even operating in N_2_ atmosphere for over 1200 h.

## 2 Result and Discussion

**Figure 1:**
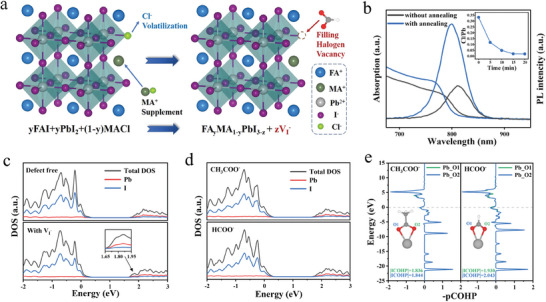
DFT simulation of the passivation effect for CH_3_COO^−^ and HCOO^−^ on the perovskite surface. a) Schematic representation of the formation of halide vacancies. b) Absorption and PL spectrum of perovskite films with annealing and without annealing. Inset: Integral area ratio of I/Pb by X‐ray photoelectron spectroscopy. The density of states (DOS) of the c): defect free, defect with *V*_I_^−^, and d): passivated CH_3_COO^−^ terminated surfaces, HCOO^−^ terminated surfaces. e) Bonding energy of CH_3_COO^−^ and HCOO^−^ with Pb^2+^ by crystal orbital overlap population simulation.

The FAPbI_3_ perovskite layer was prepared as previously reported using the One‐step solution spin coating method,^[^ ^20^ ^]^ which is regarded as “control” film in the latter. A large number of studies have shown that the halogen vacancy defects have low formation energy both in the surface and bulk of perovskite films so that the defect can easily occur during annealing treatment or air operation. The defects always serve as the center of non‐radiative recombination, which can directly affect the carrier transport between perovskite layers, thus hindering the further improvement of PSCs' performance and stability. Furthermore, we proposed another possibility of forming the halogen defects when MACl was added to the perovskite precursor solution. **Figure** **1**a shows the schematic illustration of forming halogen defects. After adding excess MACl into the perovskite precursor solution, we spin the fully dissolved solution onto the substrate and anneal at 150 °C. In this process, FA^+^ and MA^+^ ions will spontaneously combine with PbI_2_ to form FA_1‐x_MA_x_PbI_3_. However, a large amount of Cl will be volatilized in the perovskite film during the annealing process, and only a small part of Cl remains. When we square the chemical equation with Pb elements, the non‐stoichiometry of the equation results in the formation of halogen vacancies. To prove this phenomenon, the ultraviolet‐visible (UV–vis) spectroscopy and steady‐state photoluminescence (PL) spectra were employed to test the substrate without annealing and annealing film in Figure 1b. The band edge of absorption and intensity position of PL are significantly shifted, indicating the changes of perovskite bandgap after annealing, because the MA^+^ enters the A position of the perovskite structure. Besides, the PL intensity has been significantly increased, which shows that the quality of the film has been greatly improved by MACl. Then we used X‐ray photoelectron spectroscopy (XPS) to measure the peak intensity of Pb and Cl elements in two films and compared the ratio of Pb, Cl elements by integrating the areas of Pb and Cl peaks. The ratio of Cl to Pb in the wet film is ≈32.7%, corresponding to 33% MACl added into the perovskite precursor solution. And the content of Cl decreased obviously with the increasing annealing time. After annealing for 15 min, the Cl/Pb is ≈1.8% and remains unchanged. The energy dispersive spectrum (EDS) also shows a small amount of Cl element residual in annealed film in Figure S1 (Supporting Information). This proves that the overwhelming majority of Cl will evaporate with annealing, which inevitably leads to the formation of halogen vacancies. The ineluctable defect is harmful to the efficiency and stability of PSCs. In order to substantially reduce the halogen vacancy and to use the favorable effect of MACl simultaneously, two pseudo‐halogen ions were selected to fill the iodine vacancy (V_I_^−^) to achieve the passivation effect.

Hence, we first simulate V_I_^−^ in a large area of perovskite surface by the density functional theory (DFT). Figure 1c,d are the electronic structure of defect‐free, with V_I_^−^, CH_3_COO^−^, and HCOO^−^ passivated perovskite film. The presence of V_I_^−^ is accompanied by uncoordinated Pb^2+^ existing on the perovskite surface. As reported, the Pb 6p and I 5s orbitals dominate the band edges of perovskite.^[^ ^21^ ^]^ The shallow defects on the surface can affect the electron arrangement around the atom.^[^ ^22^, ^23^ ^]^ Compared with defect‐free and V_I_^−^ perovskite film in Figure 1c, the presence of V_I_^−^ will cause the defect state on the surface of perovskite, resulting in the corresponding reduction of Pb 6p electronic states, leading to significant defect energy levels (at 1.85 eV). The associated non‐radiative recombination center may directly inhibit device performance. While in Figure 1d, we simulated that CH_3_COO^−^ and HCOO^−^ can bond with exposed Pb^2+^ on the perovskite surface and fill in the V_I_^−^ position. The defect level generated by V_I_^−^ at the top of the valence band disappears and the overall electronic state is consistent with defect‐free after introducing CH_3_COO^−^ and HCOO^−^. Then, we calculate the adsorption energy (*E*_ads_)^[^ ^24^ ^]^ of CH_3_COO^−^ and HCOO^−^ adsorbed on the Pb atom in Figure S2 (Supporting Information). It can be seen that the Eads of HCOO^−^ (*E*_ads_ = −0.620) is higher than that of CH_3_COO^−^ (*E*_ads_ = −0.523). Further, the crystal orbital overlap population (COHP) calculation method was used to calculate the specific bonding energy of CH_3_COO^−^ and HCOO^−^ with Pb^2+^, as shown in Figure 1e. And Figure S3 (Supporting Information) performs entirely the schematic diagram of CH_3_COO^−^ and HCOO^−^ adsorbed on the Pb atom. It is found that two end group oxygen of CH_3_COO^−^ and HCOO^−^ will bond with the Pb atom respectively, which is labeled as O1 and O2 in Figure 1e for differentiating. The bonding energies of the two oxygen in CH_3_COO^−^ and Pb atoms are 1.836 and 1.844 respectively, which are significantly lower than that of HCOO^−^ (1.930 and 2.043). Therefore, the DFT calculation fully shows that CH_3_COO^−^ and HCOO^−^ have an obvious passivation effect on the V_I_^−^, and HCOO^−^ has better passivation effect than CH_3_COO^−^ because of its higher binding energy with Pb^2+^.

**Figure 2:**
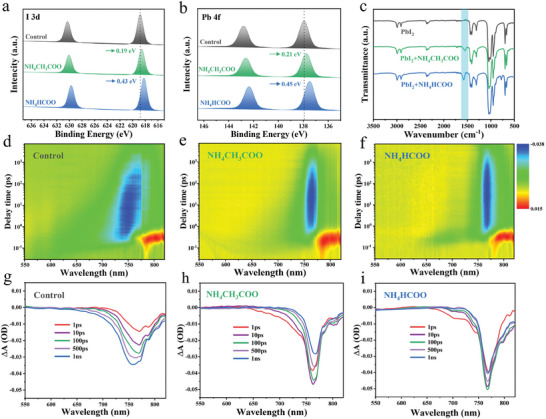
X‐ray photoelectron spectroscopy of a) Pb 4f, b) I 3d, and c) Fourier transform infrared spectroscopy for control, NH_4_CH_3_COO and NH_4_HCOO‐doped films. d–f): Transient absorption spectra at different delay times. g–i) Transient absorption line graph at delay times of 1, 10, 100, 500 ps and 1 ns.

To illustrate the passivation effects of CH_3_COO^−^ and HCOO^−^ separately, we designed volatile ammonium ions (NH_4_^+^) and selected ammonium acetate (NH_4_CH_3_COO) and ammonium formate (NH_4_HCOO) to add to the perovskite light absorption layer. The NH_4_^+^ will be converted into NH_3_ and volatilized during the annealing process, but will not remain in the perovskite film.^[^ ^25^, ^26^ ^]^ The X‐ray photoelectron spectroscopy (XPS) was conducted further to prove the bonding with CH_3_COO^−^, HCOO^−^, and Pb^2+^ in **Figure** **2**a,b, it can be seen that the main peaks of Pb 4f and I 3d for the control film located at 137.90, 142.80  and 618.80, 630.20 eV, respectively. In contrast, the binding energy of Pb 4f and I 3d all shifted to the lower binding energy by 0.21, 0.19 eV for NH_4_CH_3_COO‐doped films, and 0.45, 0.43 eV for NH_4_HCOO‐doped. The chemical shift of Pb and I peak is owing to the bonding of CH_3_COO^−^, HCOO^−^ with Pb^2+^ and indicated more electron cloud density moving toward Pb.^2+[^ ^27^, ^28^ ^]^ Then in Figure 2c, we use Fourier infrared spectroscopy (FTIR) to detect C═O stretch in NH_4_CH_3_COO and NH_4_HCOO. The stretching vibration of the C = O bond in NH_4_CH_3_COO and NH_4_HCOO is located at 1571 and 1595 cm^−1^ respectively. After testing the mixed solution of additives and PbI_2_, the vibration peaks of C═O moved to 1554 and 1570 cm^−1^, with displacements of 17 and 25 cm^−1^. This result indicates a stronger interaction between NH_4_HCOO and PbI_2_. The deviation of the C═O tensile vibration frequency in NH_4_HCOO is due to electron delocalization in oxygen after the formation of Lewis acid‐base adduct with PbI_2_.^[^ ^29^, ^30^ ^]^ This phenomenon is in accordance with DFT simulation. We conclude that CH_3_COO^−^ and HCOO^−^ anions can fill up the V_I_^−^ and bond with Pb^2+^, significantly reducing the V_I_^−^ defects in the perovskite films.

As the passivation effect of CH_3_COO^−^ and HCOO^−^ was confirmed, more specific effects on perovskite films should be studied. The NH_4_CH_3_COO and NH_4_HCOO were added to the perovskite precursor solution (for experimental details). And Figure S4 (Supporting Information) shows XRD spectra with different doping amounts (x mol.% = 1%, 2%, 3%, and 4%). In different doping concentrations, both 2% doped films obviously have the highest XRD peak value at 13.9°(100) and 27.8°(200) compared with other concentrations. The film crystallinity can be further illustrated by the reciprocal half maximum width (FWHM) in Figure S5 (Supporting Information), in contrast to the control film, the film with NH_4_HCOO shows narrower diffraction peaks and 2% doped film gets the highest value of 13.9 degree^−1^, which indicate that better crystallinity could be obtained. The ultraviolet‐visible (UV–vis) absorption of the FAPbI_3_ films (x = 1%, 2%, 3%, and 4%) was displayed in Figure S6 (Supporting Information), it clearly shows that the absorption intensity is gradually increasing from the control film to 2% doped film, corresponding to better film quality. Then, scanning electron microscopy (SEM) and atomic force microscopy (AFM) measurements were performed to directly observe the morphology of the film in Figure S7 and S8 (Supporting Information). Compared to the control film, NH_4_CH_3_COO, and NH_4_HCOO‐doped film have a slightly larger grain size from 1.0–1.2 to 1.2–1.5 um. Even though the grain size has not obviously increased, the distribution is more uniform in cross‐sectional SEM. We consider that the bonding between CH_3_COO^−^ and HCOO^−^ with Pb^2+^ can slow down the crystallization rate of perovskite, and longer crystal growth time. At the same time, the evaporation of NH_4_^+^ with annealing will lead to the upward crystallization of perovskite, which gets the vertical and “teeth‐like” grain. This “teeth‐like” grain is very favorable for carrier transport and less non‐radiative recombination. Therefore, an appropriate amount of NH_4_CH_3_COO and NH_4_HCOO doping can modulate the crystal growth processes leading to larger grain size and more uniform grain arrangement. The grain growth and good crystallization also can largely reduce the number of defects.

To investigate the transfer and recombination dynamics of the photogenerated carriers in perovskite film, transient absorption (TA) spectroscopy is conducted. Figure 2d–f exhibits typical time‐dependent 3D TA spectra of d) FTO/Perovskite, e) FTO/ NH_4_CH_3_COO‐doped and f) NH_4_HCOO‐doped perovskite film, and the images of them at different delay times can be seen in Figure 2g–i. The excitation wavelength is 480 nm, and the normalized kinetic traces of ground‐state bleaching (800 nm) are plotted. Kinetic traces are well‐fitted based on an equation:

(1) $$\Delta A\left(t\right)=-A_{0}\exp\left(-\frac{t}{\tau_{0}}\right)+A_{1}\exp\left(-\frac{t}{\tau_{1}}\right)+A_{2}\exp\left(-\frac{t}{\tau_{2}}\right)$$

where *A*_0_, *A*_1_, and *A*_2_ are the proportions of each component comprised in the total signal; *τ*_0_ is the rise time constant, *τ*_1_ and *τ*_2_ are the decay time constants, respectively.^[^ ^31^, ^32^ ^]^ The NH_4_CH_3_COO and NH_4_HCOO‐doped perovskite film shows a longer time constant (*τ*_1_ = 62 and 85 ps) compared to control perovskite film (*τ*_1_ = 39 ps) that can be attributed to the reduction of nonradiative recombination in the perovskite films. The decay time of *τ*_2_, listed in Table S1 (Supporting Information), can reflect the electron‐hole recombination.^[^ ^33^, ^34^ ^]^ The control and passivation films show 653, 873, and 1128 ps respectively, the results further indicate that there is less non‐radiative recombination caused by defect sites after NH_4_CH_3_COO and NH_4_HCOO are modified. Otherwise, the bleaching peak of the control film was apparently narrowing and redshift with delay time in Figure 2g. And no obvious variation for NH_4_CH_3_COO and NH_4_HCOO‐doped film (in Figure 2h,i), which can be attributed to the more uniform perovskite phase distribution and greatly reduced non‐radiative recombination.^[^ ^35^ ^]^ Meanwhile, Figure S9 (Supporting Information) shows the intensity of absorption peaks by fitting at the maximum bleaching wavelength. The NH_4_HCOO‐doped film has the highest absorption intensity and the fastest response time corresponding to the best film quality.

**Figure 3:**
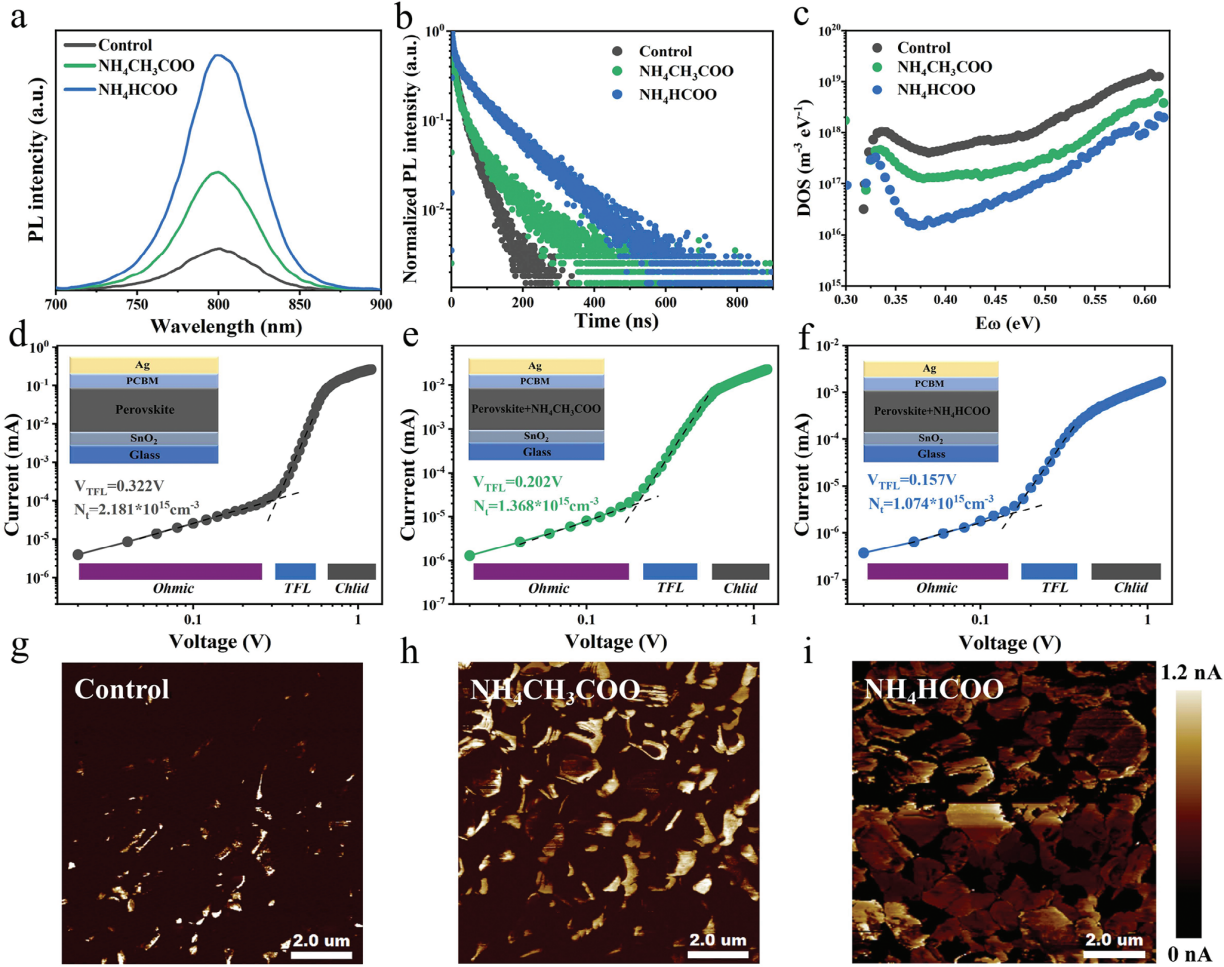
a) Steady‐state photoluminescence spectra and normalized time‐resolved photoluminescence decays b) for control, NH_4_CH_3_COO and NH_4_HCOO‐doped perovskite film. c) Thermal admittance measurement. d–f) Space charge limited current measurement for these devices. The structure is FTO/SnO_2_/perovskite/PCBM/Ag. g–i) Conductive atomic force microscopic images of control, NH_4_CH_3_COO and NH_4_HCOO‐doped perovskite film.

To illustrate the effect of NH_4_CH_3_COO and NH_4_HCOO passivation in more detail, we explored the film and device photoelectric properties of the PSCs. As shown in **Figure** **3**a, compared with the control film, the steady photoluminescence (PL) showed that the PL intensity of NH_4_CH_3_COO and NH_4_HCOO‐doped film increased remarkably, which can be ascribed to the significantly reduced V_I_^−^ defects. Figure 3b exhibited the normalized time‐resolved photoluminescence (TRPL) decays of the perovskite films deposited on the glass substrate, fitted the results by the bi‐exponential function with two components of slow decay lifetime (*τ*_1_) and fast decay lifetime (*τ*_2_).^[^ ^36^, ^37^, ^38^ ^]^ For control perovskite film, *τ*_1_(40.13 ns) and *τ*_2_ (461.72 ns), which is shorter than NH_4_CH_3_COO (68.50 ns for *τ*_1_, 830.68 ns for *τ*_2_) and NH_4_HCOO‐doped (81.53 ns for *τ*_1_, 904.90 ns for *τ*_2_) perovskite film, indicating the NH_4_HCOO doping in perovskite can greatly promote carrier transportation. Further calculation shows that the average decay lifetime (*τ*_ave_) of NH_4_HCOO‐doped perovskite film (856.78 ns) is shorter than NH_4_CH_3_COO‐doped (790.97 ns) and control perovskite film (469.63 ns), suggesting NH_4_HCOO‐doped in perovskite film would effectively increase the carrier lifetime and reduce non‐radiative recombination caused by defects.

In order to evaluate the distribution of the shallow defects and deep defects in the device, thermal admittance spectra (TAS) measurements are performed and shown in Figure 3c. The defect states can be measured by angular frequency and capacitance, and the relationship between them is derived, followed by the formula:

(2) $$N_{T}\;\left(E_{\omega }\right)=-\frac{V_{bi}}{qW}\;\frac{dC}{d\omega }\;\frac{\omega }{k_{B}T}$$

where C is the capacitance, ω is the angular frequency, q is the elementary charge, *k*_B_ is the Boltzmann constant, and T is the temperature, *V*_bi_ and W are the built‐in potential and the width of the depletion layer, respectively.^[^ ^39^, ^40^, ^41^, ^42^, ^43^ ^]^ The defect state density of the control device is ≈8.95*10^17^ cm^3^ eV^−1^ at shallow energy (0.35–0.40 eV), while the NH_4_HCOO‐doped device shows significantly reduced defect states at shallow energy level, exhibiting an order of magnitude lower than that of the control device and NH_4_CH_3_COO‐doped device, that is, the I^−^ vacancies are effectively passivated. At the same time, the defect state density at deeper energy levels also reduces, which can be attributed to better crystal growth of perovskite films after NH_4_CH_3_COO and NH_4_HCOO doping.

The typical double logarithmic *J*–*V* characteristic test can also be used to further analyze the defect state density in Figure 3d–f. The device structure is FTO/SnO_2_/perovskite/PCBM/Ag, where the perovskite film is the control, NH_4_CH_3_COO, and NH_4_HCOO‐doped perovskite film. According to formula (3), the trap‐filling limit (*V*_TFL_) determines the trap density (*N_t_*_rap_) of the device:

(3) $$\;V_{TFL}=\frac{eN_{trap}L^{2}}{2\varepsilon \varepsilon_{0}}$$

where e is the element charge, L is the thickness of the perovskite film, ε is the relative permittivity of the perovskite material, and *ε*_0_ is the vacuum permittivity,^[^ ^36^, ^44^, ^45^ ^]^ respectively. As results in Figure 3d–f, the *V*_TFL_ of the control device, NH_4_CH_3_COO, and NH_4_HCOO‐doped device is 0.322, 0.202, and 0.157 V, which is responding to the calculated defect state density of 2.181 × 10^15^, 1.368 × 10^15^, and 1.074 × 10^15^ cm^3^ eV^−1^). This result is consistent with the TAS test, strongly confirming the passivation effect of NH_4_HCOO doping. As displayed by conductive AFM (c‐AFM) to show the electrical properties of perovskite films in Figure 3g–i, the average current of control, NH_4_CH_3_COO, and NH_4_HCOO‐doped films are ≈135, 760, and 956 pA. Fewer defects lead to faster electron transport and indicate that photo‐generated carriers' local charge collection efficiency was facilitated. After adding a certain bias voltage in the AFM test, a stronger photoelectric response and improved electrical conductivity are generated.^[^ ^28^, ^46^ ^]^ It can be clearly seen that the perovskite films doped with NH_4_CH_3_COO and NH_4_HCOO have better electrical conductivity, this indicates the charge collection efficiency of photo‐generated carriers was improved, which may be due to the defects passivated by CH_3_COO^−^ and HCOO^−^.

**Figure 4:**
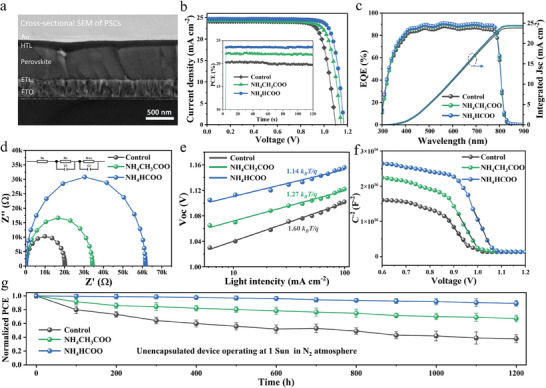
a) Cross‐sectional SEM image of PSCs. b) *J*–*V* curves of PSCs based on the control, NH_4_CH_3_COO, and NH_4_HCOO‐doped devices under reverse scan. Inset: steady PCE of the champion devices c) External quantum efficiency. d) Impedance spectroscopy measurement e) *Voc* versus light intensity and f) Mott‐Schottky measurement based on these devices. g) The efficiency of unencapsulated devices decreases after operating at the standard light in N_2_ atmosphere over 1200 h. All the error bars represent the standard deviation for six devices.

**Figure** **4**a displays the cross‐sectional SEM image of PSCs: FTO/SnO_2_/FAMAPbI_3_/ Spiro‐OMeTAD/Au. Figure 4b shows current density‐voltage (*J*–*V*) curves of PSCs based on the control, NH_4_CH_3_COO and NH_4_HCOO‐doped film under reverse scan. The champion device of NH_4_HCOO‐doped device achieved the photovoltaic parameters that open‐circuit voltage (*V*_oc_) of 1.169 V, short‐circuit current density (*J*_sc_) of 24.71 mA cm^−2^, fill factor (*FF*) of 82.13% and PCE of 23.72% that is better than that of the NH_4_CH_3_COO‐doped device (*V*_oc_ of 1.145 V, *J*_sc_ of 24.24 mA cm^−2^, *FF* of 80.10%, and PCE of 22.17%) and control device (*V*_oc_ of 1.098 V, *J*_sc_ of 24.24 mA cm^−2^, *FF* of 79.77%, and PCE of 21.23%). After NH_4_HCOO modification, the *V*_oc_ and *FF* of the device are significantly improved, which may be because the additive significantly passivates the halogen vacancy defect of perovskite, and thus reduces the non‐recombination caused by the defect during the carrier transport process inside the film, and finally shows a significant increase in *V*_oc_ and *FF*. To illustrate the universality of this strategy, we made 30 devices to test their efficiency, respectively. The reverse scanning efficiency and distribution of 30 devices are shown in Figure S10 (Supporting Information). It can be observed that NH_4_HCOO‐doped devices have excellent results, and the overall efficiency is significantly higher than the control devices. The steady PCE at their MPP reveals the same trend consistent with performance promotion (inset). Furthermore, compared to the hysteresis index of 0.107 of control devices, the NH_4_CH_3_COO and NH_4_HCOO‐doped devices show negligible hysteresis of 0.064 and 0.016 (Table S3, Supporting Information). The external quantum efficiency (EQE) spectra (Figure 4c) also showed that the value of *J*_sc_ of PSCs was 24.41 mA cm^−2^ for the NH_4_HCOO‐doped device, 24.13 mA cm^−2^ for the NH_4_CH_3_COO device and 23.97 mA cm^−2^ for control device, which is well matched with the measured value from the *J–V* results.

The electrochemical impedance spectroscopy (EIS) measurement was performed to explore the recombination during charge transport. The Nyquist plots and equivalent circuits are displayed in Figure 4d and the fitting parameters are listed in Table S4 (Supporting Information). The equivalent circuits represent series resistance (*R*_s_), transport resistance (*R*_tr_), and recombination resistance (*R*_rec_) with capacitors (*C*_1_ and *C*_2_),^[^ ^47^, ^48^ ^]^ respectively. It indicated that the resistance at high frequency is 18.3 Ω for the control device, 29.4 Ω for the NH_4_CH_3_COO‐doped device, and 43.5 Ω for the NH_4_HCOO‐doped device. The resistance at low frequencies is 20005, 35290, and 61864 Ω for these devices. These results revealed higher recombination resistance for the NH_4_HCOO‐doped device, which is in accordance with the higher *Voc* and FF.

To further analyze charge carrier recombination and transportation, the dark *J*–*V* and *Voc*‐light intensity measurements were conducted for these devices. As shown in Figure S11 (Supporting Information), the dark current density based on the NH_4_HCOO‐doped device was lower than the control and NH_4_CH_3_COO‐doped device, indicating a reduced leakage current that is due to the enhanced shunt resistance. And Figure 4e reveals the relationship between the measured *V*_oc_ and light intensity for the reference and NH_4_HCOO‐doped PSCs, the slope of the fitting line corresponding to the m*k_B_T/q*, where the m is ideality factor, *k*_B_ is the Boltzmann constant, q is the electron charge and T is the temperature. The value of m can reflect the nonradiative recombination between layers.^[^ ^20^ ^]^ The m of the control device is 1.60, 1.27 for the NH_4_CH_3_COO‐doped, and 1.14 for the NH_4_HCOO‐doped device. It reveals the less nonradiative recombination with the passivation effect of NH_4_HCOO. Moreover, the Mott‐Schottky measurement was used to evaluate the built‐in potential (*V*_bi_) in Figure 4f, compared these devices, the *V*_bi_ for the control device is 1.03, 1.05 V for NH_4_CH_3_COO‐doped and 1.10 V for NH_4_HCOO‐doped. The increase of the *V*_bi_ further indicates that defect reduction promotes carrier transport and less non‐radiative recombination at the interface.^[^ ^49^, ^50^ ^]^

Then, we studied the universality of pseudo‐halogen treatment under different MACl contents and selected the addition of 10%, 20%, and 30% MACl respectively. After adding 2% NH_4_HCOO, the device's efficiency was significantly improved, especially for the improvement of FF and Voc. Table S5 (Supporting Information) shows the specific photoelectric parameters. The modified device's improved efficiency with different MACl contents shows the method's universality.

The stability of the solar cell is also a key factor in evaluating the performance of the device. Figure 4g displays the stability of unencapsulated devices after operating under continuous 1‐sun illumination in N_2_ atmosphere over 1200 h. The NH_4_HCOO‐doped devices could retain ≈90% of their initial PCE, exhibiting excellent stability than NH_4_CH_3_COO and control devices. The poor stability of the control device may be due to the internal defects of perovskite leading to more carrier recombination during operation, while the defects of the NH_4_HCOO‐doped device are largely suppressed, thus obtaining excellent efficiency and operational stability.

## 3 Conclusion

In summary, we proposed for the first time that the addition of MACl leads to a non‐stoichiometric ratio of perovskite, and the resulting perovskite would inevitably produce halogen vacancies due to the non‐synchronous volatilization of methylamine cations and chloride ions. Therefore, two pseudo‐halogen anions (CH_3_COO^−^ and HCOO^−^) were selected to modify the generated vacancy defects. The DFT simulation and experimental results together confirm that CH_3_COO^−^ and HCOO^−^ can fill the *V*_I_^−^ position to form the bond with Pb^2+^. This strategy can effectively eliminate the formation of *V*_I_^−^ and uncoordinated Pb^2+^ defects in the perovskite films. In addition, HCOO^−^ has a better passivation effect than CH_3_COO^−^ because of its higher binding energy with Pb^2+^. Based on the 2% mole ratio of NH_4_CH_3_COO and NH_4_HCOO doping, the PCE of the champion devices can be improved from 21.23% to 23.72% with negligible hysteresis. And the unencapsulated device can still retain >90% of the initial PCE even operating in N_2_ atmosphere for over 1200 h. This work illustrates another halogen defect source in the MACl‐assisted formamidine perovskite photovoltaics and provides a new route to obtain high‐performance perovskite solar cells.

## Conflict of Interest

The authors declare no conflict of interest.

## Supporting information

Supporting Information

## Data Availability

Research data are not shared.
