# Supplementary material for: Eliminating Halogen Vacancies Enables Efficient MACL‐Assisted Formamidine Perovskite Solar Cells
Source: Adv Sci (Weinh). 2023 Dec 8;11(7):2306280. doi: 10.1002/advs.202306280 (PMC10870047; doi:10.1002/advs.202306280)
Supplement: Supplementary file 1 — Supporting Information [file ADVS-11-2306280-s001.pdf]

ADVANCED SCIENCE
Open Access

Supporting Information

for *Adv. Sci.*, DOI 10.1002/advs.202306280

Eliminating Halogen Vacancies Enables Efficient MACL-Assisted Formamidine Perovskite Solar Cells

Zhiyong Liu, Tianxiao Liu, Meng Li, Tingwei He, Gaofu Guo, Pengfei Liu, Ting Chen, Jien Yang, Chaochao Qin, Xianqi Dai and Mingjian Yuan**

Supporting Information

1. Experimental Section

1.1 Chemical and materials

FTO conductive glass with a square resistance of 15 Ω/sq was purchased from Advanced Electron Technology CO, Ltd. The tin oxide electronic layer material was prepared by diluting the 15% tin oxide colloidal dispersion of Alfa Aesar company by 3 times. Lead iodide (PbI_2, 99.99%), formamidine iodide (FAI, ≥ 99.5%), and Methylammonium chloride (MACl, ≥ 99.5%) were purchased from TCI and Great Cell Solar, respectively. 2,2',7,7'-Tetrakis[N, N-di(4-methoxyphenyl)amino]-9, 9'-spirobifluorene (Spiro-OMeTAD) and additives (TBP, Li-TFSI) were bought from Xi'an Polymer Light Co., Ltd. N, N-dimethylformamide (DMF, 99.8%), dimethyl sulfoxide (DMSO, 99.9%), chlorobenzene (99.9%) was purchased from Sigma-Aldrich. Ammonium acetate and ammonium formate (99.5%) was purchased from Macleans.

1.2 Device fabrication

The laser-etched conductive FTO substrate was continuously washed in an ultrasonic bath using detergents, ethyl alcohol, acetone, and isopropanol for 15-20 min, respectively. Before use, the FTO substrates were dried with nitrogen and treated in an ultraviolet-ozone system for 30 min. The SnO_2 colloidal dispersion (1:3, v/v in ultrapure water) was spin-coated onto the FTO substrate at 3000 rpm for 30 s. Subsequently, the SnO_2 layer was annealed at 150 ℃ for 30 min in air. After that, the substrate with the SnO_2 layer was transferred into the glovebox filled with N_2. For preparing the $FAPbI_3$ precursor solution, 1.4 M FAI and PbI_2 with NH_4HCOO and NH_4CH_3COO at different molar ratios (0%, 1%, 2%, 3%, and 4%) were added in a 4:1 mixture of DMF and DMSO. Then 0.45 M MACl was added to support the formation of the black phase. The perovskite solution was spin-coated on the substrate at 5000 rpm for 30 s. The antisolvent of 140 ul EA was dripped on the film at 25 seconds after the start of spin coating. Then the wet film was annealed at the glove box at 220℃ for 5 seconds and transferred out from the glovebox to anneal at 150 ℃ for 15 min in the air with a relative humidity of 20-30%. After that, the PEAI (5

mg/ml in IPA) solution was spin-coated onto the film at 4000 rpm for 30 s in the glove box. Subsequently, Spiro-OMeTAD solution (72.3mg Spiro-OMeTAD powder, 28.8μL 4-tertbutylpyridine and 17.5 μL Li-TFSI solution (520 mg Li-TFSI in 1 mL ACN) in 1 mL CB was spin-coated on the substrate at 5000 rpm for 30 s. Finally, a 100 nm Au electrode was deposited on the substrate by thermal evaporation under a high vacuum. A mask with an active area of 0.064 cm^2 was employed.

1.3 Device characterization

The field emission scanning electron microscope (FESEM, Zeiss Supra55) was used to characterize the surface and cross-sectional morphology of the perovskite film. Furthermore, the EDS system equipped with scanning electron microscope can measure the element distribution of perovskite. The more detailed surface morphology of perovskite films was obtained with atomic force microscope (AFM: Asylum Research MFP-3D-Stand Alone). The photovoltaic performance of the device is measured by 2400 source meters under AM 1.5G simulated sunlight. EQE was tested by a Newport QE measurement kit. X-ray photoelectron spectroscopy (XPS) was conducted on a Thermo ScientificTM K-AlphaTM$^+$ spectrometer equipped with a monochromatic Al Kα X-ray source (148.66 eV) operating at 100 W. Samples were analyzed under vacuum ($P < 10^{-8}$ mbar) with a pass energy of 150 eV (survey scans) or 50 eV (high-resolution scans). The XPS spectra were calibrated by the binding energy of 284.8 eV for C 1s. Bruker D8 ADVANCE X-ray diffractometer was used to determine the crystallinity of the perovskite film; The UV-Vis spectrophotometer (Agilent Cary 5000) was used to collect the absorbance spectra of the perovskite films; The PL and TRPL spectra were conducted with a fluorescence spectrophotometer (Edinburgh instruments FLS 980) using a Xe lamp and 450 nm excitation source, respectively. The defect state density of the perovskite device was evaluated by thermal admittance spectroscopy (TAS).

2. DFT Computational Details

All DFT calculations were performed using the spin-polarized Vienna ab initio simulation package (VASP) code. The exchange-correlation functional was described by generalized gradient approximation (GGA) method in the form of Perdew-Burke-Ernzerhof (PBE). The cut-off energy was set to 500 eV, and the vacuum space in the z-direction is set to 20 Å to prevent periodical effects. The Γ-centered Monkhorst-Pack K points of 3×3×1 and 7×7×1 were applied for geometry optimization and electronic properties. In addition, the convergence ranges for energy and force are set to 10^{-5} eV/atom and 0.01 eV/Å, respectively, throughout the calculation.

In particular, based on the formula

$$E_{ads} = E_{adsorbate+sub} - E_{adsorbate} - E_{sub}$$

Where $E_{adsorbate+sub}$, $E_{adsorbate}$, and E_{sub} are the total energies of the adsorbate-substrate the isolated adsorbate, and the substrate, respectively. Thus, a negative E_{ads} indicates exothermic adsorption.

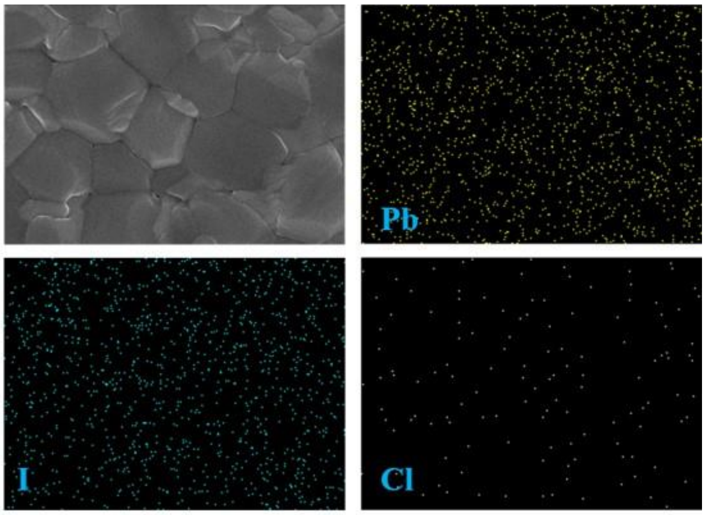

Figure S1 EDS spectra (Pb, I, Cl element) of annealing film with MACl addition

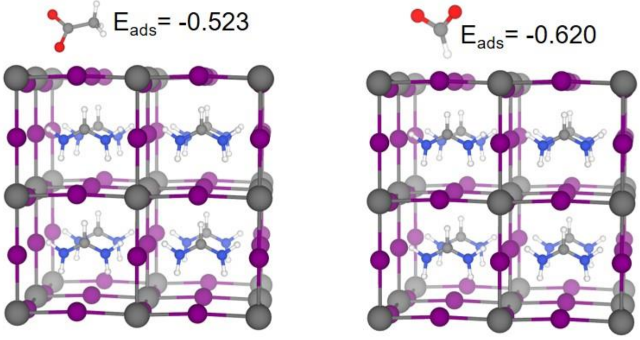

Figure S2 The E_{ads} energy of CH_3COO^- and $HCOO^-$ ions adsorbed on the Pb atom.

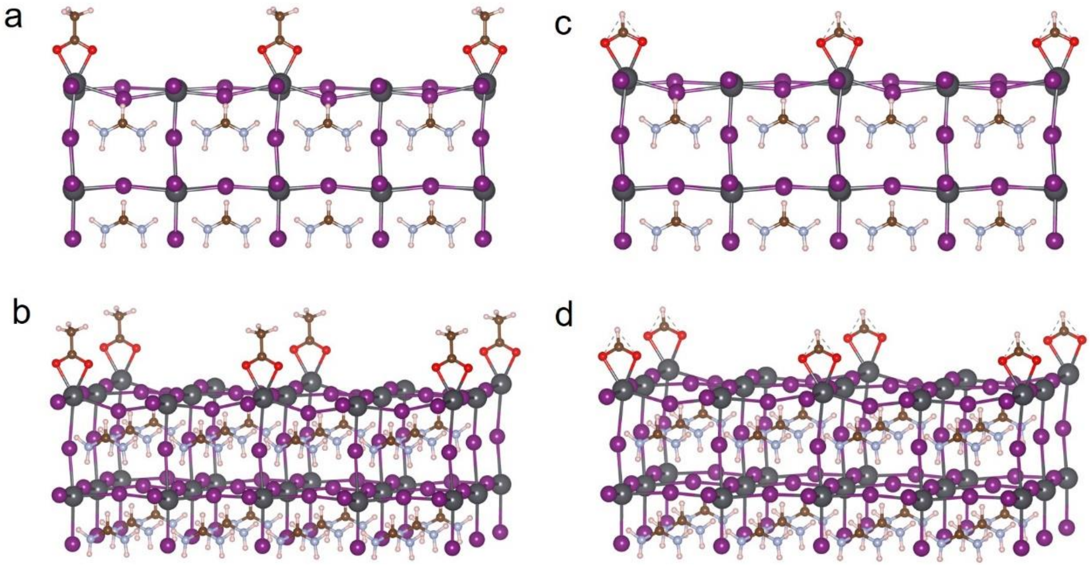

Figure S3 Schematic diagram of CH_3COO^- and $HCOO^-$ ions adsorbed on the Pb atom

in DFT simulation.

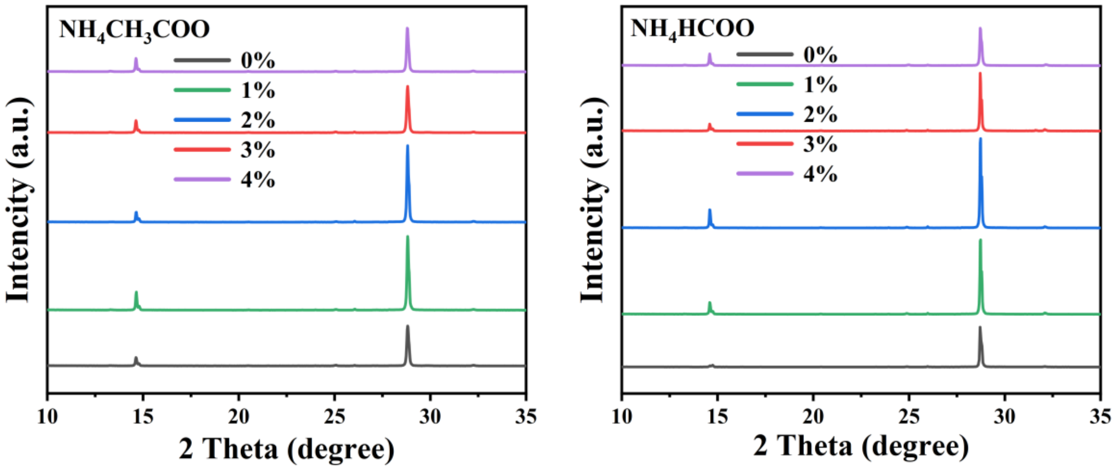

Figure S4 XRD spectra of perovskite film with 1-4% NH_4CH_3COO and NH_4HCOO doping.

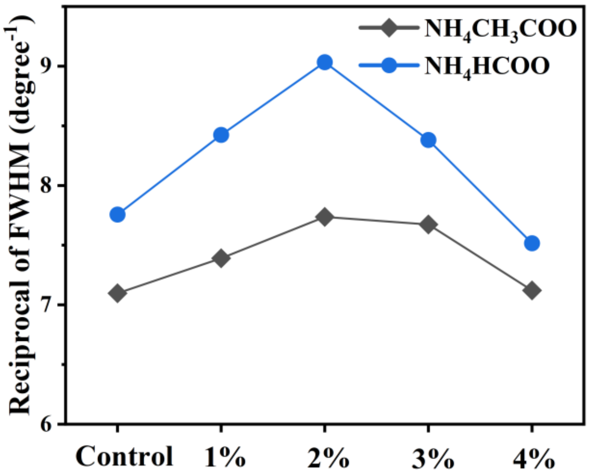

Figure S5 The reciprocal of FWHM by XRD spectra.

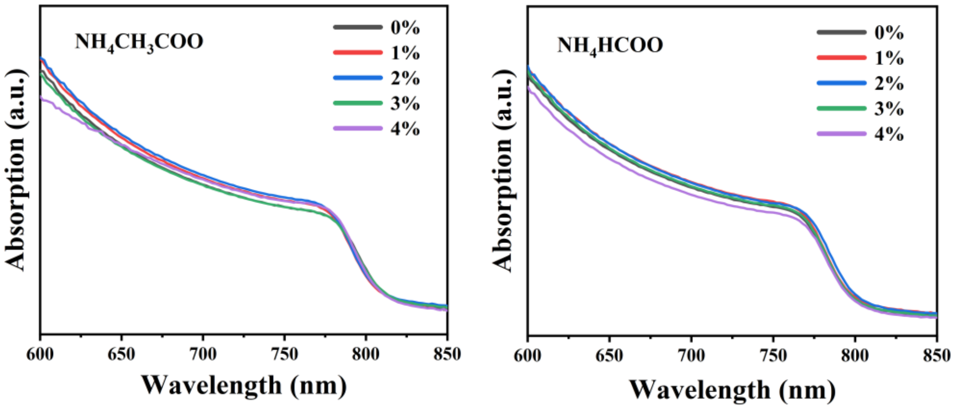

Figure S6 UV-vis absorption of perovskite film with control and 1%, 2%, 3%, 4% NH_4CH_3COO and NH_4HCOO-doped.

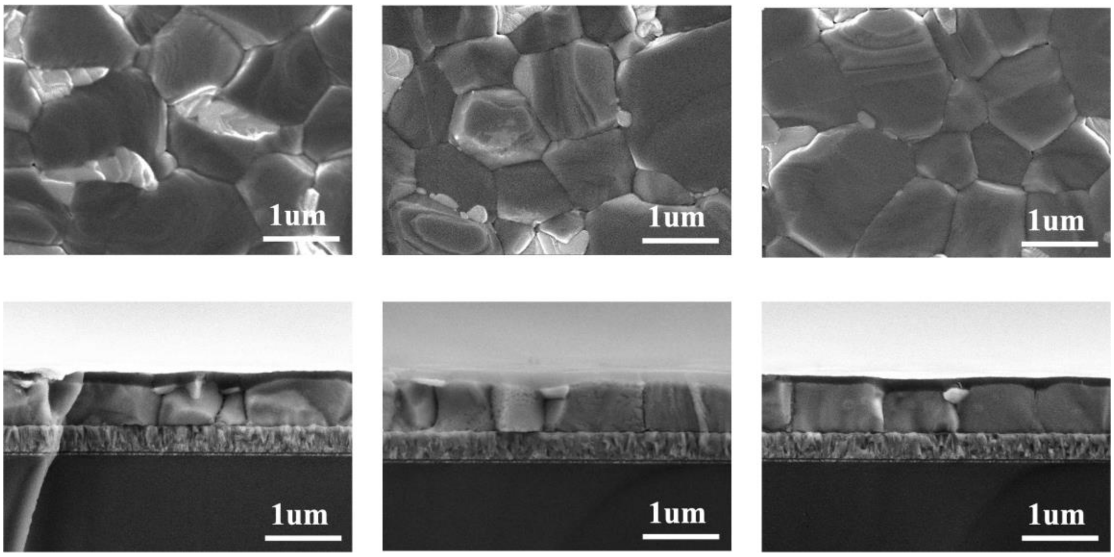

Figure S7 Surface and cross-section SEM images of control, NH_4CH_3COO, and NH_4HCOO-doped films.

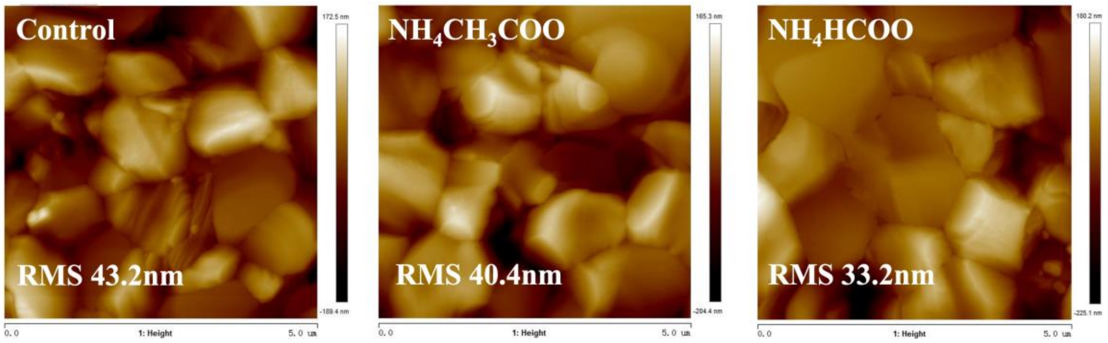

Figure S8 The atomic force microscopy images of Control, NH_4CH_3COO, and NH_4HCOO films.

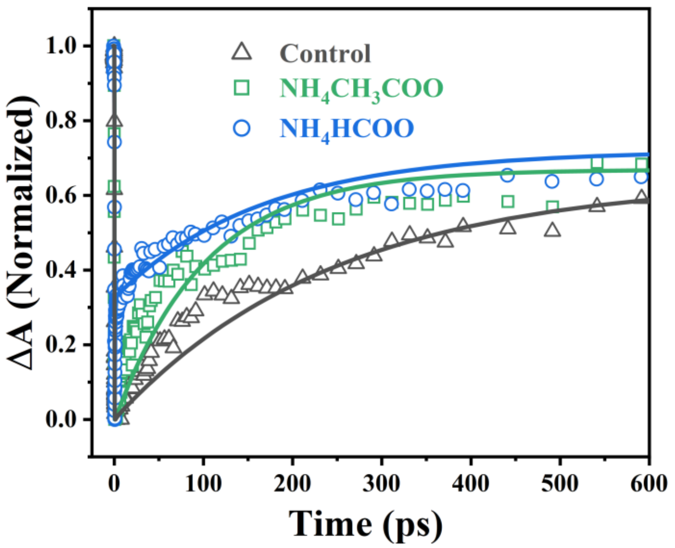

Figure S9 The fitting of femtosecond spectrum at the maximum bleaching peak.

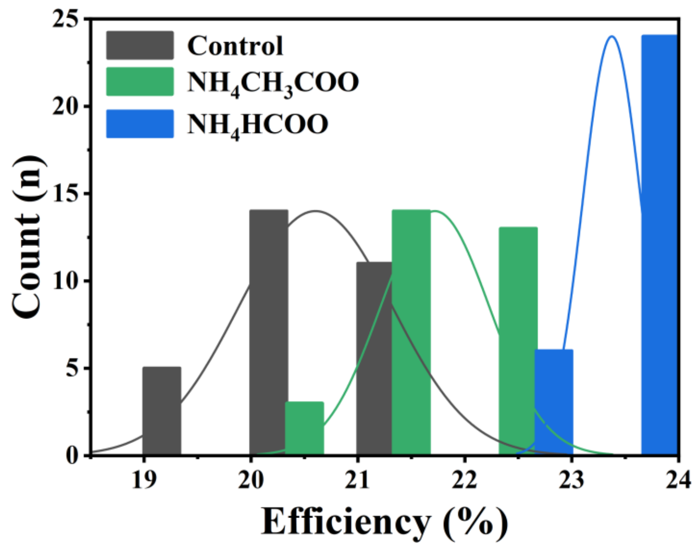

Figure S10 The highest efficiency and 30 devices' efficiency distribution of Control, NH_4CH_3COO, and NH_4HCOO-doped devices.

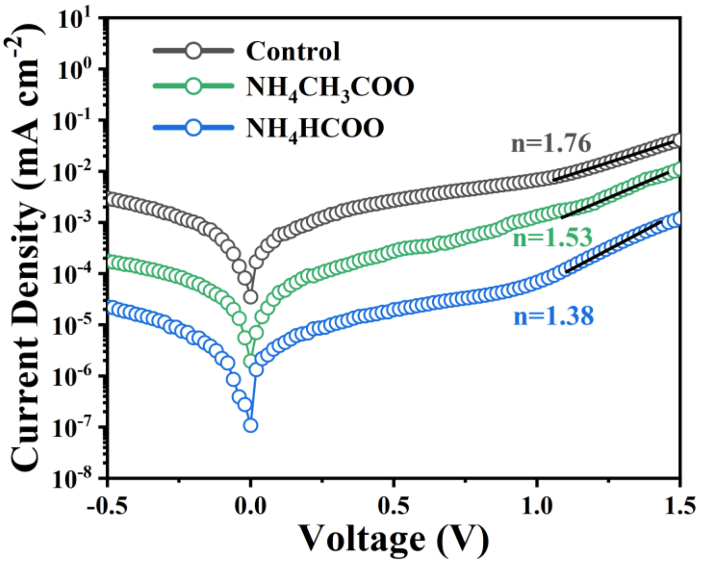

Figure S11 Dark J-V Curves of Control, NH_4CH_3COO, and NH_4HCOO-doped films

	A_1	τ_1 (ps)	A_2	τ_2 (ps)	τ_{avg}
Control	0.28	39	0.43	653	346
NH_4CH_3COO-doped	0.32	62	0.48	873	467.5
NH_4HCOO-doped	0.37	85	0.52	1128	606.5

Table. S1 Kinetic fit of transient absorption spectroscopy parameters of control, NH_4CH_3COO, and NH_4HCOO-doped perovskite film.

	A_1	τ_1 (ns)	A_2	τ_2 (ns)	τ_{avg}
Control	0.21	40.13	0.33	461.72	469.63
NH_4CH_3COO	0.30	68.50	0.45	830.68	790.97
NH_4HCOO	0.36	81.53	0.49	904.90	856.78

Table. S2 TRPL parameters of control, NH_4CH_3COO, and NH_4HCOO-doped perovskite films.

Device	Direction	V_{OC} (V)	J_{SC} (mA cm^{-2})	FF (%)	PCE (%)	*H-index*
Control	RS	1.098	24.24	79.77	21.23	0.107
	FS	1.068	24.23	72.92	18.95	
NH_4CH_3COO	RS	1.145	24.24	80.10	22.17	0.064
	FS	1.120	24.21	76.74	20.74	
NH_4HCOO	RS	1.169	24.71	82.13	23.72	0.016
	FS	1.158	24.72	81.53	23.33	

H-index is calculated by an equation: H-index = (PCE_{RS} − PCE_{FS})/PCE_{RS}.

Table. S3 The optimal photoelectric parameters and hysteresis index of control, NH_4CH_3COO, and NH_4HCOO-doped perovskite device in forward and reverse scan.

Device	R_s (Ω)	R_{tr} (Ω)	R_{rec} (Ω)
Control	18.3	229.6	20005
NH_4CH_3COO	29.4	140.0	35290
NH_4HCOO	43.5	98.7	61864

Table. S4 Impedance spectroscopy parameters of the perovskite cells with and without NH_4CH_3COO, and NH_4HCOO passivation.

Device	V_{OC} (V)	J_{SC} (mA cm^{-2})	FF (%)	PCE (%)
10%MACl	1.062	24.04	75.20	19.20
NH_4HCOO	1.085	24.23	77.85	20.46
20%MACl	1.118	24.18	75.12	20.31
NH_4HCOO	1.125	24.27	77.86	21.26
30%MACl	1.135	24.51	78.40	22.81
NH_4HCOO	1.157	24.62	80.96	23.06

Table. S5 Comparison of photoelectric parameters of devices with different MACl contents by NH_4HCOO-doped.
